# Modulated Modularity Clustering as an Exploratory Tool for Functional Genomic Inference

**DOI:** 10.1371/journal.pgen.1000479

**Published:** 2009-05-08

**Authors:** Eric A. Stone, Julien F. Ayroles

**Affiliations:** 1 Department of Statistics, North Carolina State University, Raleigh, North Carolina, United States of America; 2 Bioinformatics Research Center, North Carolina State University, Raleigh, North Carolina, United States of America; 3 W. M. Keck Center for Behavioral Biology, North Carolina State University, Raleigh, North Carolina, United States of America; 4 Department of Genetics, North Carolina State University, Raleigh, North Carolina, United States of America; Princeton University, United States of America

## Abstract

In recent years, the advent of high-throughput assays, coupled with their diminishing cost, has facilitated a systems approach to biology. As a consequence, massive amounts of data are currently being generated, requiring efficient methodology aimed at the reduction of scale. Whole-genome transcriptional profiling is a standard component of systems-level analyses, and to reduce scale and improve inference clustering genes is common. Since clustering is often the first step toward generating hypotheses, cluster quality is critical. Conversely, because the validation of cluster-driven hypotheses is indirect, it is critical that quality clusters not be obtained by subjective means. In this paper, we present a new objective-based clustering method and demonstrate that it yields high-quality results. Our method, modulated modularity clustering (MMC), seeks community structure in graphical data. MMC modulates the connection strengths of edges in a weighted graph to maximize an objective function (called modularity) that quantifies community structure. The result of this maximization is a clustering through which tightly-connected groups of vertices emerge. Our application is to systems genetics, and we quantitatively compare MMC both to the hierarchical clustering method most commonly employed and to three popular spectral clustering approaches. We further validate MMC through analyses of human and *Drosophila melanogaster* expression data, demonstrating that the clusters we obtain are biologically meaningful. We show MMC to be effective and suitable to applications of large scale. In light of these features, we advocate MMC as a standard tool for exploration and hypothesis generation.

## Author Summary

Systems genetic approaches integrate classical methods with transcriptional profiling and other modern assays to make inference at the network level. It is customary to partition the genes entering such an analysis into clusters destined for independent interrogation, but there is a danger of facilitating a hypothesis that is falsely self-fulfilling. Motivated by the dual issues of scale and subjectivity, we present a new clustering method designed to elicit transcriptional modules from gene expression profiles that is both effective and automatic. Modulated modularity clustering (MMC) seeks community structure in graphical data—in this case, a graph of genes connected by edges whose weights reflect the degree to which transcriptional profiles correlate. MMC modifies this graph to make communities stand out and returns the clustering that describes this community structure. We begin with a numerical study to show that MMC is able to recover community structure from simulated data. We then demonstrate similar success on biological data by obtaining human and *Drosophila* gene clusters that, in each case, are intuitive and biologically meaningful. We advocate the use of MMC as an exploratory tool for functional genomic inference. A Web server for MMC is available at http://mmc.gnets.ncsu.edu.

## Introduction

With the diminishing cost of high-throughput biological assays, the generation of large and multifaceted datasets has become commonplace. Scale, once limiting, is now a feature to be exploited, and researchers have recognized implications beyond an increased sample size. The classical reductionist approach to biology, and to genetics in particular, has begun to cede ground to a systems view in which complex interactions supplant single loci as the units of study. Today, systems genetic approaches integrate classical methods with transcriptional profiling and other modern assays to make inference at the network level [1]. However, while early successes have illuminated networks of genes responsible for complex traits and human disease, the underlying inference is inherently challenging [2],[3],[4]. Networks expand the scope of traditional analysis dramatically: 10,000 genes become 100 million gene pairs that may interact to varying degrees, and this is before considering directionality or higher-order relationships. Thus, scale has become an issue once again, only now the limitation is computational. A second issue is validation; experimentally testing systems hypotheses is difficult at best, and often validation comes indirectly through multiple forms of corroborating evidence. While it is necessary to manage scale and desirable to facilitate validation, simultaneously addressing these concerns is precarious. It is customary to partition the genes entering a systems genetic analysis into clusters destined for independent interrogation [5],[6],[7],[8]. Incorporating subjective criteria into this clustering step is natural, but when the rubric is indirect validation, there is a danger of facilitating a hypothesis that is falsely self-fulfilling.

This study is motivated by the dual issues of scale and subjectivity. We consider the problem of clustering similar transcriptional profiles and propose an approach that is both effective and automatic. Our method, *modulated modularity clustering* (*MMC*), is explicitly designed to elicit latent structure (i.e. communities) from weighted graphs, and we demonstrate that the communities identified by MMC are predictive of coherent transcriptional modules. Moreover, the approach we describe is objective-based and self-consistent: the complete clustering is identified by maximizing a single measure of community structure over all possible gene partitions, with no interference from tuning parameters or external validation. As a prelude to applications, we begin with a discussion of community structure, of the measure used by MMC to quantify it, and of the methodology from which that measure is derived.

The goal of clustering is to classify objects into some number of groups such that objects within a group are similar while objects in different groups are not [9]. The idea of community structure is related, except that similarity is described by the edges connecting vertices in a graph. Newman [10] describes community structure in a network as a statistically surprising arrangement of edges. Thus, a community is a cluster of objects (vertices) whose aggregate similarity (edge set) exceeds random expectation. Likewise, genes that comprise a community feature transcriptional profiles that are in aggregate surprisingly correlated. The idea of clustering transcriptional profiles is not new [11],[12], nor is the idea of interrogating such data for community structure [13],[14]. What distinguishes our approach is its ability to resolve meaningful community structure in the face of heterogeneous similarity measured on a continuous scale; the precise scenario that results from computing correlations between transcriptional profiles.

**Figure 1:**
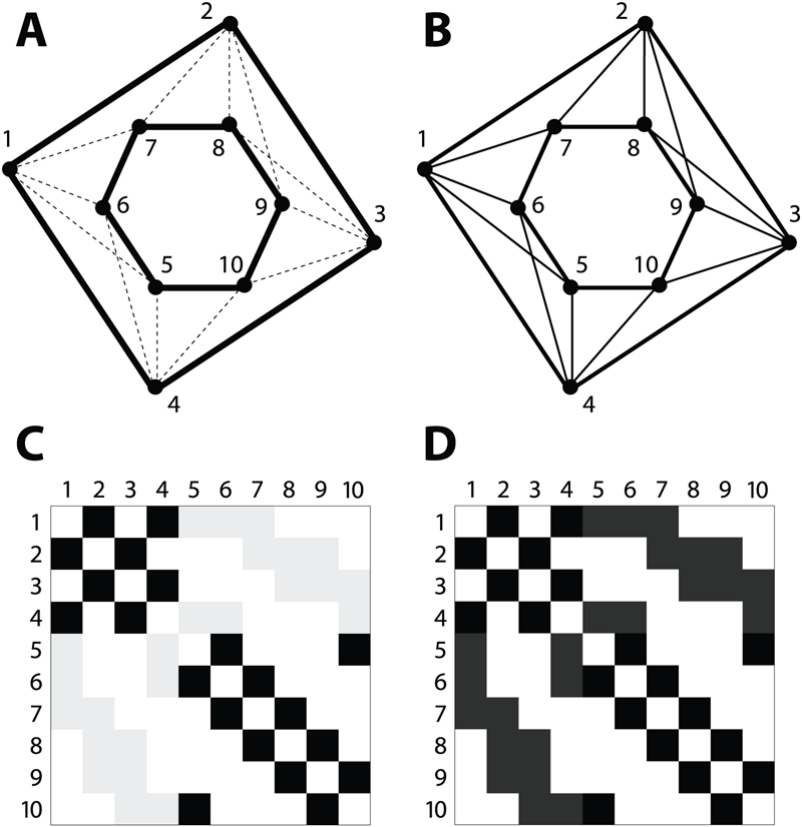
Community structure in graphs and affinity matrices. (A) A graph with 10 vertices and 22 edges. Thick black lines denote strong edges and thin dotted lines denote weak edges. The edge classes have been drawn so that the distinction between them is prominent: {1,2,3,4} and {5,6,7,8,9,10} are intuitive communities of connected vertices. (B) Ostensibly the same graph as in the previous panel, with the edge classes drawn to obscure the communities that were so prominent. (C) Depiction of the affinity matrix that corresponds to the graph in panel (A). Rows and columns denote vertices, and each (row,column) entry of the matrix is shaded to indicate the strength of its corresponding edge, if any. As drawn, the contrast between strong and weak edges is sufficient to reveal two communities as clusters of darkly shaded squares along the matrix main diagonal. (D) Depiction of the affinity matrix that corresponds to the graph in panel (B). With less contrast between edge classes, the pattern along the main diagonal is largely obscured.

MMC uses the concept of modularity [15] to quantify community structure. Defined for an unweighted graph, the modularity of any partition (clustering) measures the difference between the total number of edges connecting vertices that share a cluster and what would be expected in an equivalent graph with edges placed at random [10],[15],[16]. Thus, when modularity is greater than zero, the similarity between clustered vertices exceeds random expectation, which is an intuitively desirable quality for a clustering to have. Unfortunately, intuition breaks down when the edges of a graph are weighted. In this case, the partition of maximum modularity may not be that which is most desirable, as edge weight heterogeneity can yield trivial clusters dominated by a handful of extreme values. MMC addresses this, but we are not the first to propose a solution; in [17], a rank-based transformation is applied to the edges, resulting in an unweighted graph where only the most strongly connected vertex pairs remain connected. Clearly this discards a great deal of useful information, but more importantly, undesirable properties emerge. Such is the case in the graph of Figure 1A, which shows ten vertices connected by edges that are either strong (thick lines), weak (thin lines), or nonexistent. Visual inspection clearly indicates two clusters ( {1,2,3,4} and {5,6,7,8,9,10}), but this grouping is invisible to the rank-based approach of [17]. By contrast, MMC is able to elicit this community structure, both in Figure 1A where the distinction between edge weights is dramatic and in Figure 1B where that distinction is subtle.

The idea of MMC is to modulate the spectrum of edge weights parametrically by means of a nonlinear transformation. Visually, Figure 1B becomes Figure 1A for the purposes of detecting community structure, with the result that even subtle communities are revealed. Analytically, MMC includes an additional parameter 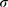 into the modularity objective function (see Materials and Methods), and the joint maximum over all partitions 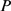 and values of 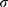 is sought. It is clear that the optimal partition changes as 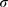 varies: in the graph of Figure 1B, for example, modularity dictates that there is no community structure when 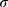 is large, whereas when 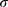 is small, the two clusters so prominent in Figure 1A emerge. Because the modularity obtained when 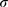 is small is greater than that obtained when 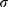 is large, MMC clusters the vertices into {1,2,3,4} and {5,6,7,8,9,10} rather than report that no community structure was found.

In what follows, we generate a small example dataset and use it to illustrate the method of MMC step by step. We then demonstrate the performance of MMC on both real and simulated data, and in the process make direct comparisons with hierarchical clustering and three graph-based spectral methods. Though MMC perceives data as graphical, our discussion is presented in terms of matrices. Specifically, each weighted graph can be represented by an affinity matrix whose rows and columns represent vertices and whose entries are the edge weights between vertex pairs. Thus, the graphs indexed by 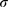 that MMC considers can also be viewed as a parametric family of affinity matrices, and each of these matrices can be illustrated succinctly. As an example, consider what is shown in Figures 1C and 1D. Here the graphs from Figures 1A and 1B, respectively, have been illustrated as affinity matrices, with grayscale used to emulate line thickness. In this scheme, it is clear that contrast can either reveal or obscure the pattern. By analogy, it is useful to consider each of the forthcoming results as structure that manifests once MMC has determined the optimal level of contrast.

## Results

The nature of clustering is such that it is difficult to make objective comparisons between methodologies. Thus, in this section we have chosen to focus mainly on demonstrating the effectiveness of MMC as a tool for biological inference. We first illustrate the method on a small simulated dataset for which it can be argued that a “correct” clustering exists. In this case, we do quantitatively compare MMC's performance to that of four other clustering methods. We then turn to two biological examples, demonstrating how MMC can be used to predict coherent transcriptional modules both from the gene expression profiles of 40 wild-derived, inbred lines of *Drosophila melanogaster* and from 1,240 individual expression profiles obtained from human blood samples. Here we cannot say what is correct, but we provide multiple sources of external biological evidence that link the transcripts assigned to a cluster.

### Modulated Modularity Clustering by Example

**Figure 2:**
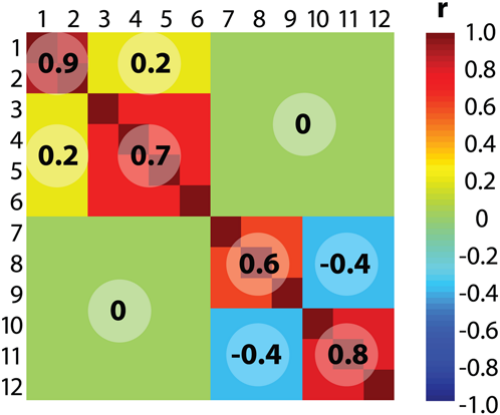
Correlation structure for simulated data. Twelve variables are correlated as shown in the figure. The heat map used to illustrate the pairwise correlations ranges from dark red (perfect correlation, 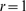) through green (no correlation, 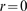) to dark blue (perfect anticorrelation, 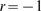). On the diagonal are four correlated clusters of varying strength: {1,2} with an 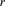 of 0.9, {3,4,5,6} with an 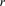 of 0.7, {7,8,9} with an 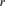 of 0.6, and {10,11,12} with an 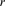 of 0.8. There are nonzero correlations between two pairs of clusters; members of {1,2} and {3,4,5,6} are positively correlated (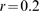), while members of {7,8,9} and {10,11,12} are negatively correlated (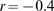).

**Figure 3:**
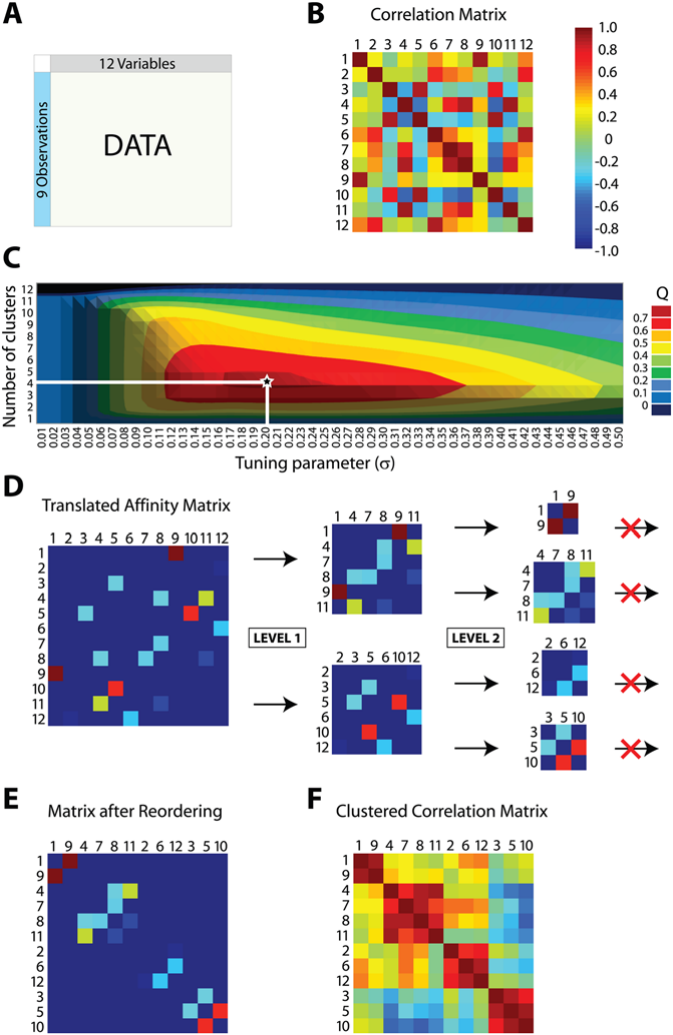
Modulated modularity clustering. Panels (A)–(F) illustrate the method of modulated modularity clustering by example. (A) Example data consisting of nine observations on twelve variables drawn from a standard multivariate Normal distribution with variance-covariance matrix given by the correlation matrix from Figure 2. The variables have been permuted so that the block structure of the data will be obscured. (B) Heat map showing the pairwise correlations between variables (numbered 1 through 12 *after* permutation). The color scheme was introduced in Figure 2. (C) Surface plot of the maximum modularity attainable for a fixed number of clusters as 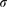 varies. The 4,213,597 possible partitions of the twelve variables are grouped by number of parts (and hence clusters), and the maximum modularity 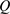 found among these is shown on the plot for each 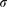. The surface appears convex and attains its maximum 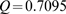 at 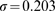 (on a grid of step size 0.001) for a clustering of size 4. For larger examples, it is not possible to enumerate all partitions, and an approximate method is used to marginally maximize 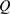 in 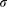. (D) The optimal 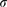 defines the graph whose community structure is to be evaluated. The graph has affinity matrix with entries 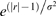; for illustration, these values have been shifted and linearly scaled so that the previously introduced heat map applies. To identify the partition of maximum modularity, we use a greedy forward search in which the initial graph is recursively bisected into subgraphs until the overall modularity can no longer increase. In the figure, each level (LEVEL 1, LEVEL 2) indicates a round of bisection, and each subgraph is represented by its corresponding section of the affinity matrix. There is no third level; subsequent to the second round, the overall modularity cannot be increased through further bisection. (E) The resulting clustering is used to reorder the affinity matrix by permutation of its rows and columns. Entries with colors other than dark blue have now been aggregated along the main diagonal. (F) Applying the same permutation to the correlation matrix reveals the four correlated clusters of variables ({1,9},{4,7,8,11},{2,6,12},{3,5,10}) hidden within the data.

We begin with a simulated dataset composed of nine observations drawn from a 12-dimensional multivariate Normal distribution whose variance-covariance matrix includes four correlated components (shown in Figure 2). These dimensions were chosen both for ease of illustration and so that an exhaustive search for the optimal clustering was feasible (as shown in Figure 3C). Figure 3 depicts the flow of our simulated data through MMC, beginning with a depiction of the raw data matrix as input in Figure 3A. As shown in Figure 3B, the data are interpreted from their 12×12 matrix of pairwise Pearson product-moment correlations between variables. Here and in subsequent figures we rely on a heat map to visualize the range of values from −1 to +1; the colors range from dark red (+1, perfect correlation) through green (0, no correlation) to dark blue (−1, perfect anti-correlation). In MMC, the correlation matrix from Figure 3B gives rise to a continuum of weighted graphs and associated affinity matrices parameterized by 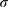. The goal is to find the partition 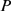 and value of 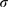 that jointly maximize the modulated modularity objective function 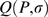. Ideally, this search can be conducted simultaneously; in practice, we first seek an approximate solution to the joint maximization to obtain a value of 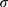 and then marginally maximize over 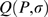 with 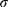 fixed (see Materials and Methods). To illustrate how 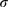 is obtained, for our small example we have rendered the exact maximization surface of 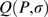 in two dimensions as Figure 3C. The horizontal axis of the plot specifies 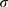 and determines the graph from which modularity is calculated, while the vertical axis indexes the 4,213,597 possible partitions of the twelve variables, grouped by number of parts *k* (and hence number of clusters). At the intersection of 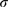 and 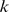, the plot shows the maximum modularity attainable for those fixed values; as indicated in Figure 3C, the joint maximum modularity of 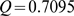 is attained at 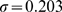 for a partition with 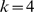. For datasets of even modest size, the exact maximization surface is intractable, and we resort to fast approximations to obtain the optimal value of 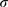 without specific regard to obtaining the optimal 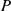. The result is an optimal affinity matrix, shown for our simulated data in Figure 3D. Note that the affinity matrix takes values between 0 and 1 inclusive and has zeros on the diagonal, implying that it corresponds to an undirected, weighted graph with no loops. For the sake of illustration, we have translated the range of the affinity matrix to [−1, 1] so that the heat map introduced in Figure 3B is applicable. With the optimal value of 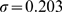, the pairwise correlations between variables have been protracted so that those of the largest magnitude are emphasized. The strongest correlations from Figure 3B (dark red off-diagonal entries) now span the entire range of [−1, 1], while those of lesser magnitude are reduced to near negligibility (see also Figure S1). In essence, the edge weights of the optimal graph have been modulated to best emphasize the community structure relating the variables; all that remains is to enumerate community membership in the form of clusters. Because in our implementation 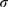 is now fixed, the MMC objective function reduces to that of Newman and Girvan's modularity, and we can use any of the techniques already developed for its maximization [10],[18],[19],[20],[21]. We have chosen Newman's iterative bisection approach because we have found it to work well in practice [10]. The approach is illustrated in the remainder of Figure 3D. We first seek the bipartition of maximal modularity using a two-step procedure in which an approximate solution is locally refined (e.g. Level 1, see Materials and Methods). We then iterate, splitting the resultant parts (e.g. Level 2) in a greedy attempt to further maximize the overall modularity. As shown in the figure, for our simulated data the bipartition of maximum modularity groups the twelve variables into {1, 4, 7, 8, 9, 11} and {2, 3, 5, 6, 10, 12}. Each part from Level 1 is subjected to further splitting, yielding four parts in Level 2: {1, 9}, {4, 7, 8, 11}, {2, 6, 12}, and {3, 5, 10}. Additional splitting is now fruitless – any further division actually decreases the overall modularity – and the procedure terminates with these four clusters. Having defined the clusters, Figure 3E reconstructs a permuted affinity matrix in which the rows and columns have been reordered so that the clustered variables are contiguous. Figure 3F shows the associated correlation matrix, similarly permuted so that the clusters of correlated variables are now obvious.

### Validation and Comparison by Simulation

In the simulated data example of Figure 3, MMC recapitulates the four latent components perfectly. It is clear, however, that the results may change upon clustering nine new observations drawn from the same multivariate Normal distribution. To place the performance of MMC in some context, we repeatedly sampled datasets of nine observations from the multivariate Normal distribution previously described. For each of these datasets, we recorded the results of MMC, as detailed above, and of average linkage agglomerative hierarchical clustering, using the same distance function as was used in MMC. We chose this form of hierarchical clustering for comparison because of its prevalence, particularly in applications to gene expression data [5],[22],[23],[24],[25]. To enrich the comparison, we also considered the performance of three spectral clustering methods. In each, we used the optimal affinity matrix 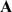 determined by MMC to construct a graph Laplacian 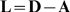 where 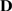 is the diagonal matrix whose entries are the row sums of 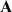. The unnormalized version of spectral clustering to which MMC was compared operates on the eigenvectors of 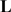. For a prespecified number of clusters 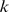, we extracted into a matrix the eigenvectors of 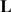 corresponding to its 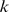 smallest eigenvalues. To achieve a spectral clustering, the rows of this matrix were viewed as points in 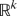 and clustered with 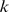-means. Motivated by Shi and Malik [26], we also repeated this procedure for the normalized Laplacian 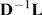. Lastly, we considered the variant introduced in [27] that clusters based on the symmetric normalized Laplacian 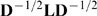. Here, before clustering with 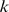-means, we standardized the extracted matrix of eigenvectors so that each row had unit norm.

Thus, our simulation study compared the performance of MMC to that of four other methods. Because each method to which MMC was compared leaves the number of clusters 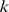 to be specified (or otherwise determined), we structured the simulation in parts. We began by considering how the competing methods perform when they are seeded with a realistic but incorrect number of clusters, in this case three. Across 10,000 simulated datasets, we scored all five methods for each simulation by recording which pairs of variables were correctly clustered (or separated) and which were not. Assuming that only variables from the same correlated component in Figure 2 should be clustered together, we calculated the proportion of simulations in which each pair of variables was aligned correctly. The results, reported in Table 1, show MMC to be more accurate than its competitors (85.6% vs. less than 80%) when these competing methods seek a reasonable but suboptimal number of clusters. More convincingly, Table 1 also shows MMC to be superior when all five methods are informed of the correct number of clusters, four. To assess performance in this setting, we again simulated datasets under the same distributional assumptions; this time, however, we restricted our consideration to only those cases in which MMC found four clusters. For 10,000 such cases we compared MMC to its competitors, and as before MMC was the most accurate among the five methods considered (91.5% vs. less than 89%). As Table 1 reports, MMC was superior both at clustering pairs of variables meant to be clustered and at separating those meant to be separated.

**Table 1:** Comparison of Clustering Methods over 10,000 Simulated Datasets.

Method	Variant	%Correctly Clustered	% Correctly Separated	% Correct
**Modulated Modularity**	*Default*	**82.4%**	**86.4%**	**85.6%**
	*k* = 4	**81.0%**	**94.1%**	**91.5%**
**Agglomerative Hierarchical**	*k* = 3	84.4%	76.1%	77.7%
	*k* = 4	79.5%	89.8%	87.8%
**Unnormalized Spectral**	*k* = 3	82.6%	68.6%	71.3%
	*k* = 4	76.0%	83.4%	82.0%
**Normalized Spectral**	*k* = 3	78.2%	70.8%	72.2%
	*k* = 4	70.0%	84.9%	81.9%
**Symmetric Spectral**	*k* = 3	81.1%	78.9%	79.4%
	*k* = 4	77.1%	91.8%	88.9%

**Figure 4:**
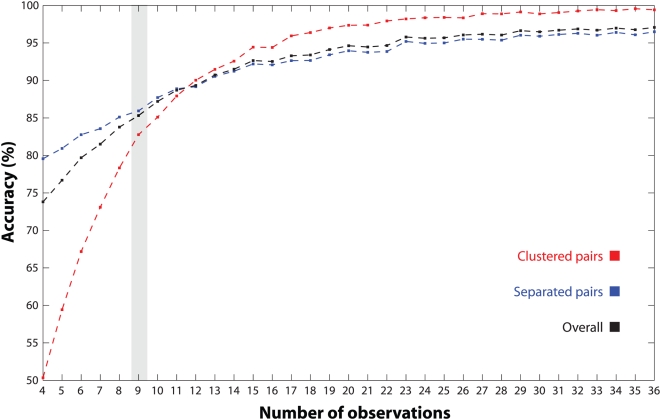
MMC analysis of simulated data. Three measures of accuracy are reported for MMC as the number of observations ranges from 4 to 36 in the simulation study. Shown in red for each case is the percentage of simulations in which pairs of variables are correctly clustered. Shown in blue is the percentage of pairs correctly separated; the overall percentage correct is in black. Each point on the plot represents the results across 1,000 datasets. The scenario considered in Table 1 (MMC default) is highlighted in gray.

Five clustering methods are compared in two simulation studies. Studies are grouped by row, so that the same 10,000 simulated datasets were used to evaluate default MMC and the four remaining methods with 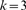. A second set of 10,000 datasets was used to compare the methods with 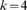. Columns 2–4 report three related measures of performance. Column 2 considers only pairs of variables that share a cluster according to Figure 2 (e.g. (1, 2) but not (1, 3)) and records the percentage of such pairs that are correctly clustered together. Column 3 considers only pairs of variables that do not share a cluster and records the percentage of such pairs that are correctly placed in separate clusters. Column 4 considers all variable pairs and records the overall percentage correct. These measures are used in Figure 4 as well.

Beyond providing a measure of accuracy, the results of Table 1 are indicative of confidence and cluster stability. Under the conditions of our simulation study, MMC frequently (and correctly) clustered the same variables together, and we observe this phenomenon more generally when resampling. Indeed, the same quantity we report in Table 1 can be used to summarize a collection of bootstrapped MMC clusterings [28], though we have not presented such an analysis here. It is clear that both sample size and cluster structure impact MMC; we chose to investigate how the former influences sampling variation by varying the number of observations in our previous simulation study. Whereas before we clustered twelve variables from nine observations, here we considered sample sizes ranging by ones from four to thirty-six. In each case, we simulated 1,000 datasets and used the performance measures from Table 1 to evaluate MMC. The results, shown in Figure 4, indicate that while four observations are sufficient to reveal cluster structure, each additional observation greatly helps resolve clustered variable pairs. This trend continues for increasing sample size but with diminishing returns; as we have seen, with nine observations MMC already performs quite well. Thus, at least in our example, MMC is able to resolve cluster structure in rank deficient data. We have observed that it does so better than four competing methods, with the significant feature that for MMC the number of clusters need not be prespecified or otherwise arbitrarily ascertained.

### Systems Genetic Analysis of *Drosophila melanogaster* Data

**Figure 5:**
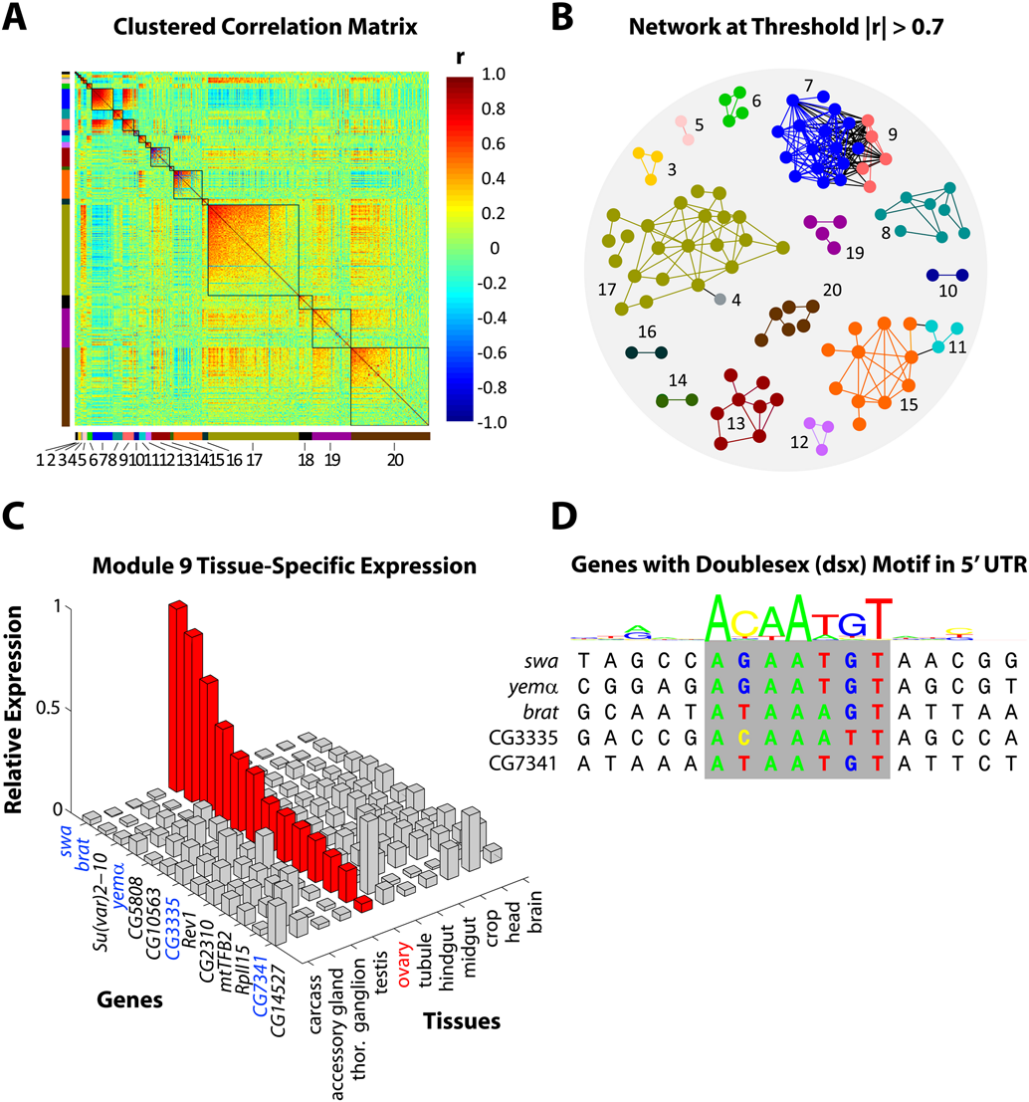
MMC analysis of *Drosophila melanogaster* data. Panels (A)–(D) describe a systems genetic analysis of 414 *Drosophila melanogaster* genes associated with a competitive fitness phenotype. (A) The reordered matrix of pairwise genetic correlations between transcriptional profiles, in analogy to Figure 3F. The twenty clusters identified by MMC are numbered (Modules 1–20), color-coded (to the left and below), and emphasized with borders. From the upper left (Module 1) to lower right (Module 20), modules are ordered by decreasing average connectivity, defined here as average absolute pairwise correlation within the module. (B) Relevance network obtained from the 414 genes by enforcing an absolute correlation threshold of 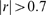. The genes are numbered and color-coded as in (A) to indicate module membership. Only genes with at least one connection are shown. (C) Bar chart of the genes in Module 9 reporting for each one its relative expression level across eleven tissues. Genes are shown on the 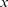-axis, tissues are shown on the 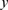-axis, and relative expression is shown on the 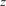-axis. Expression in the ovary has been highlighted in red, and the genes featured in the next panel have been highlighted in blue. (D) Local alignment of five genes sharing the *dsx* motif in their 5′ UTRs. Above the alignment, a logo is shown to represent the profile of the 17 bp recognition sequence of *doublesex*.

Having demonstrated the efficacy of MMC on simulated data, we now turn to a biological example. Our data come from a recent study of 40 highly-inbred lines of *Drosophila melanogaster* derived from the Raleigh, NC, natural population [2]. Here we focus on the transcriptional profiles of 414 genes whose expression levels were found to significantly associate with a phenotypic measure of competitive fitness. A summary of the experiment and data collection is provided in Materials and Methods; details can be found in [2]. As shown in Figure 5A, MMC identifies twenty clusters of correlated transcriptional profiles among the 414 fitness-associated genes. The clusters range in size from 2 to 106 and, significantly, each represents a putative transcriptional module (henceforth Modules 1–20) comprised of genes that are genetically intercorrelated across the 40 inbred *Drosophila melanogaster* lines. Often, genes whose transcriptional profiles covary over time or treatment are represented as connected nodes in an interaction or relevance network (e.g. [29]); in Figure 5B, we have done the same for genes whose transcriptional profiles are correlated across lines. Specifically, we color-coded the twenty modules from Figure 5A and superimposed them in Figure 5B onto the graph obtained by connecting genes whose absolute genetic correlation was above an arbitrary threshold of 0.7. As the figure shows, the connected components are largely homogeneous in terms of cluster membership, suggesting that MMC is automating what might reasonably result from manual curation (e.g. using Cytoscape [30]). We emphasize, however, that the intuitive clustering produced by MMC was done automatically without resorting to hard thresholding or external tuning parameters. More importantly, the putative transcriptional modules identified as clusters by MMC are biologically meaningful. As reported in [2], we identified modules enriched for genes that mediate immune response (Modules 6 and 11), visual perception and function of the nervous system (Module 17), chemosensation (Module 20), and for sex-specific transcripts (Modules 7, 8 and 9). To draw contrast, we note that the hierarchical clustering approach considered in the simulations above can be also be used to obtain 20 modules here; doing so groups the transcripts in such a way that the sex-specificity that characterizes Modules 7, 8, and 9 is obscured. In what follows, for the sake of brevity, we have chosen to elaborate the biological relevance of only Module 9. Of the thirteen genes that comprise Module 9, six encode predicted transcripts of unknown function. The remainder, as indicated in Figure 5C, include *swallow*, *brain tumor*, *suppressor of variegation 2–10*, *yemanuclein α*, *Rev1*, *mitochondrial transcription factor b2*, and *RNA polymerase II 15kd subunit*. Our transcriptional profiling of the genes in Module 9 revealed a pattern of female-biased expression [2], and an independent source of tissue-specific *Drosophila* expression data identified these genes as being highly expressed in the ovary [31]. The latter is shown in Figure 5C; for each gene in Module 9, the figure reports its expression level in each of eleven tissues as a fraction of the total observed across all tissues. Thus, Module 9 is characterized by female-biased genes that are highly expressed in the ovary, and further elucidation can be found through sequence analysis of the untranslated regions upstream of each gene. We downloaded the 5′ UTR of each gene in Module 9 from FlyBase [32] and searched for the presence of any of 62 *Drosophila* transcription factor motifs. In doing so, we identified the *doublesex* (*dsx*) motif as being significantly overrepresented (*P*<0.001), appearing in the 5′ UTR of five genes in the module. Figure 5D shows the motif sequences of the five genes that share *dsx* in their 5′ UTRs as well as the canonical profile of the 17 bp recognition sequence. Figure 5C indicates that three of the five genes shown (*swallow*, *brain tumor*, and *yemanuclein*) were also among the top genes in terms of relative expression in the ovary. *Doublesex* is a transcription factor that regulates sexual differentiation in *Drosophila* [33], and sequence-based evidence that it regulates the genes in Module 9 complements our observations of female-biased and ovary-enhanced expression. Thus, using MMC as our starting point, we now have a basis for annotating the six unknown genes in Module 9 as well as for a candidate biological process in which all thirteen genes may be involved. Though we have limited our discussion to Module 9, other modules suggest hypotheses that are equally compelling [2]. We view this as support for MMC as a method for obtaining meaningful clusters from biological data; conversely, we believe that the objective-based approach of MMC bolsters the biological hypotheses founded upon it.

### Analysis of Human Lymphocyte Data

**Figure 6:**
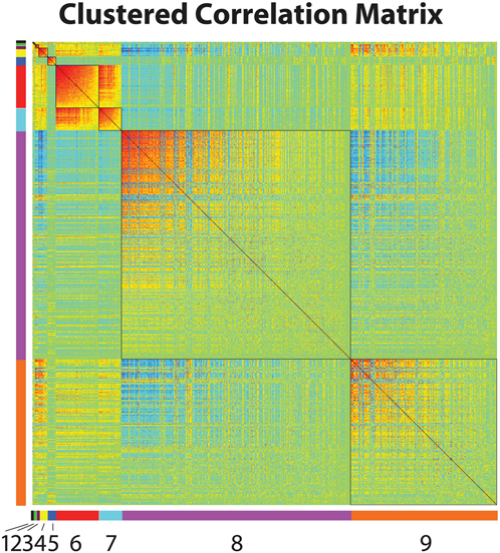
MMC analysis of data from the San Antonio Family Heart Study. Clustering of a set of 673 transcripts associated with HDL-C concentration; shown is the reordered matrix of pairwise correlations between transcriptional profiles. The nine clusters identified by MMC are numbered and arranged as in Figure 5.

**Figure 7:**
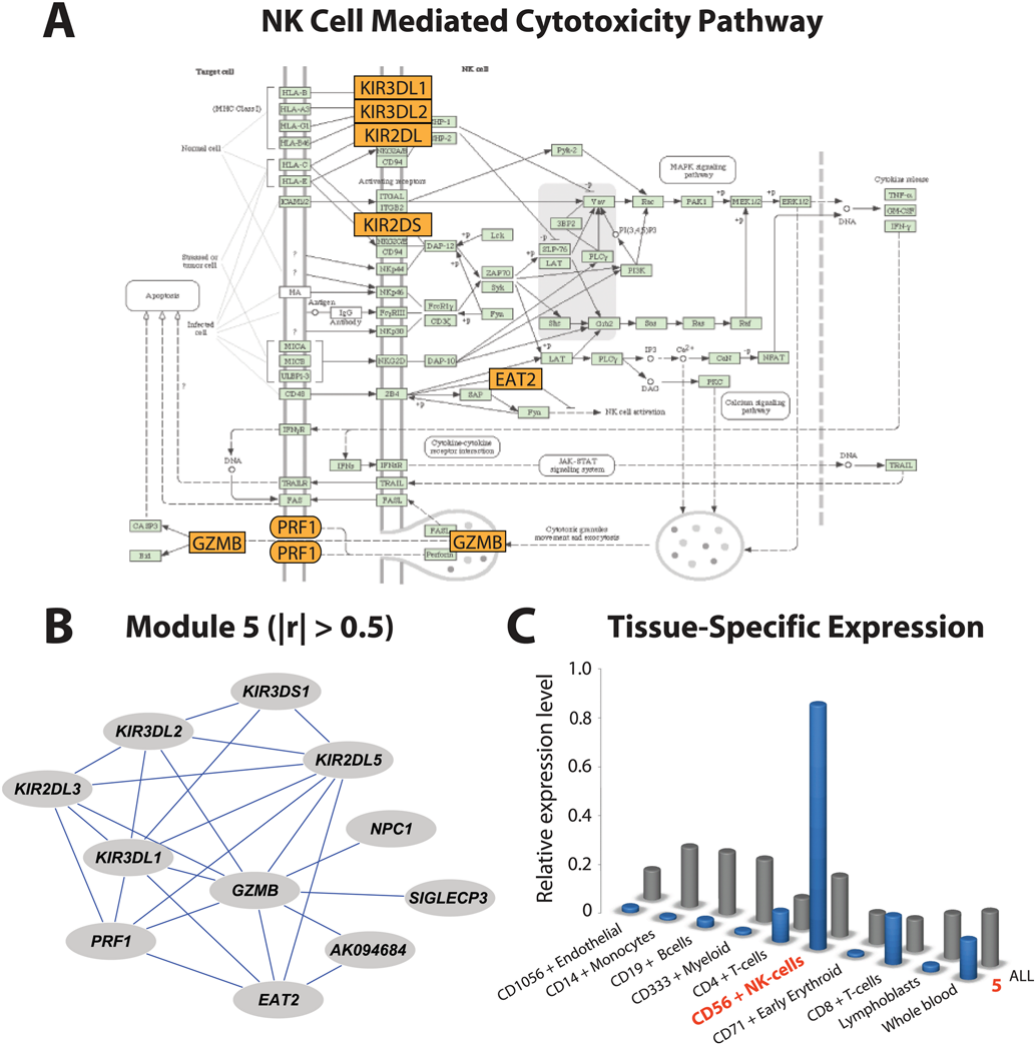
Illustration of module-specific enrichment of pathways and tissues. (A) Representation of the NK cell mediated cytotoxicity KEGG pathway. Genes in Module 5 associated with HDL-C variation are colored orange. (B) Relevance network representation of Module 5 after enforcing an absolute correlation threshold of 0.5. (C) Bar graphs of cell-specific expression patterns restricted to derivatives of whole blood. Grey bars indicate relative cell-specific expression levels across all 673 genes associated with HDL-C. Blue bars consider only the genes in Module 5.

As a final demonstration of MMC's utility, we turned to a human dataset generated by the San Antonio Family Heart Study [34],[35] In this study, genome-wide transcriptional profiling was performed on 1240 individuals using lymphocyte extracted from blood samples. For each individual, age and sex were recorded as covariates, and high density lipoprotein (HDL-C) concentration was measured. More details about the experimental design are given in [34]. These data offer the opportunity to identify transcripts associated with HDL-C; to that effect, we constructed linear regression models for each expressed transcript including the effects of both age and sex. We uncovered 673 genes significantly associated with variation in HDL-C levels at a 0.05 FDR. Proceeding as in the *Drosophila* example above, we then used MMC to cluster these genes into nine modules of correlated transcripts (Figure 6). We next asked to what extent these hypothesized transcriptional modules mapped to known pathways or were enriched for particular biological processes. Considering the 673 HDL-C-associated genes as statistical background, we used DAVID [36] to assess for each module the degree to which biological processes and pathways were overrepresented. We found that Module 3 is involved in translation; 80% of the genes in this module are components of the small ribosomal subunit (P = 1.20E-06, 1.70E-04 corrected). Likewise, Module 5 is highly enriched for genes involved in natural killer (NK) cell mediated cytotoxicity (Figure 7A; P = 7.40E-11, 9.40E-09 corrected) and Module 6 is enriched for members of the B cell receptor signaling pathway (P = 2.80E-04, 3.50E-02 corrected).

Though our observations ultimately require validation at the bench, there is ample evidence to indicate that the genes clustered here by MMC have known interactions relevant to the general function of their cluster. Module 5 is of particular interest – while there is a well characterized relationship between NK cell activity and HDL-C levels [37] the underlying functional genomic basis of this relationship does not appear to be known. This module contains a number of membrane receptor and transports (Figure 7A,B), including five genes encoding killer cell immunoglobulin-like receptors (*KIR2DL1*, *KIR2DL5A*, *KIR2DS5*, *KIR3DL1*, *KIR3DS1*), *granzyme B* (also known as *Natural killer cell protease 1*), *perforin 1* (which in culture medium increases endocytosis of granzyme B protein [38]), *EAT2* (which suppresses NK cell activation), and *SIGLECP3* (which encodes an NK-cell-specific transmembrane protein [39]). As verified by SymAtlas [40], each of these genes is expressed primarily in NK cells, and this pattern is pervasive within but specific to Module 5 (Figure 7C). We interpret this observation as strong support for MMC, but it also raises an unexpected possibility: the correlated expression patterns in Module 5 may be an artifact of individual variation in NK cell count among the lymphocytes extracted. We lack the data to interrogate this possibility directly, but it is worth noting that Module 5 contains relevant genes whose expression patterns are not thought to be cell-type biased; one such example, *Niemann-Pick type C* (*NPC1*), encodes a protein that mobilizes unesterified cholesterol from the lysosomal compartment to the intracellular sites where it can be metabolized or excreted in NK cells [41],[42]. In light of the association between Module 5 gene expression patterns and concentrations of HDL-C, if the correlated patterns we observe are indeed an artifact, then NK cell count presents a biologically interesting confounder.

## Discussion

In this paper, we present a novel clustering method with applications to transcriptional profiling. Our method, MMC, builds upon the concept of modularity to quantify the extent of community structure present in a weighted graph, largely without regard to how the edge weights have been initially calibrated. Our motivation was to elicit transcriptional modules from the genetic correlations between expression profiles of 40 wild-derived, inbred lines of *Drosophila melanogaster*. The results suggest that the clusters produced by MMC are coherent and biologically meaningful.

MMC was developed in response to a specific set of concerns. First, while there is a vast body of work on clustering algorithms, only a small fraction of the literature is dedicated to the problem of community structure. We envisioned transcriptional modules as tight communities within a completely connected graph of the transcriptome and developed MMC to identify these. Second, we sought to balance the information available in the strengths of pairwise relationships against the possibility that these strengths might be uncalibrated. The statistical distinction between genetic correlations, for example, is a function of both magnitude and sample size; we developed MMC to adaptively modulate the magnitude of pairwise relationships in the search for maximal community structure. Third, we wished to avoid specifying the number of clusters in advance. Because MMC does not view more clusters as necessarily being better, clusterings of different sizes can be compared with impartiality. Fourth, we did not want to resort to external criteria to determine a proper number of clusters or to specify a minimum cluster size. Whereas other procedures use peripheral measures such as the “elbow” criterion, cluster silhouettes [43], or a gap statistic [44],[45] to choose the number of clusters, MMC weighs clusterings of all sizes consistently under the same objective function used to establish cluster membership. Fifth, we did not want to introduce tuning parameters or opportunities for user-defined thresholds. MMC is fully automated and independent of the application.

As a graph-based procedure, MMC shares features with other clustering approaches that seek optimal cuts of graphs. The list of objective functions used in such approaches continues to grow and includes the normalized cut, the ratio cut, and the modularity criterion from which MMC's is derived. Moreover, because we have chosen an eigenvector-based approach to optimize the MMC objective function, our specific implementation can be classified as spectral. Indeed, the aforementioned spectral clustering approaches to which MMC was compared can also be seen as relaxed solutions to objective functions for cutting graphs. As we have highlighted MMC's automated ability to choose the number of clusters, it should be noted that the spectra of graph Laplacian matrices are at least informative in that regard. The eigengap heuristic, for example, is a principled mechanism for choosing an appropriate number of clusters [46]. Nevertheless, such criteria are again external to the clustering procedure, whereas MMC seeks and defines the optimal clustering based on a single objective function.

The applications presented in this paper range from small (12 simulated variables) to intermediate (414 *Drosophila* genes, 673 human genes) in scope. Because the algorithm we use to maximize 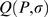 is fast, results for our simulated data were returned almost instantaneously, while the *Drosophila* and human analyses took less than fifteen minutes on a typical desktop computer. Though not optimized for speed, we have found our Matlab implementation of MMC to be suitable for even datasets of very large size. For example, using data from the same 40 highly-inbred *Drosophila melanogaster* lines as previously described, in [2] we clustered 10,096 genetically variable transcripts into 241 transcriptional modules. Here our implementation of MMC required several days for completion, but because the search for 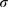 is easily parallelized, run time can be reduced considerably. Our code is freely available, requires only a data file as input, and generates results along with figures similar to that shown in Figure 5A. In light of its effectiveness and ease of use, we envision MMC as a standard tool for exploration and hypothesis generation.

## Materials and Methods

### Distance and Similarity Metrics

We have used the absolute correlation 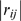 to define the raw similarity between vectors of observations 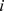 and 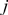. From this we defined the Euclidean-like pairwise distance metric 
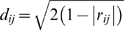
.

### Modulated Modularity

As an optimization criterion, modulated modularity is a parametric extension of the modularity concept first proposed by Girvan and Newman [15]. Given an undirected graph 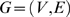 with affinity matrix 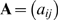, each partition 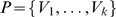 of the vertex set 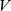 yields the *modularity* 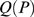 given by
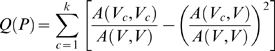
where we use 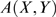 to denote the sum of the entries in the submatrix of 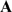 whose rows and columns are indexed by the vertices in 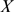 and 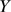, respectively. *Modulated modularity* extends the application of modularity to weighted graphs by introducing a monotone transformation of the edge weights 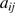. We use a one-parameter family of monotone functions to modulate the difference in strength between edge weights so that a highly structured graph emerges. Following the recommendation of [27], we use a Gaussian transformation to define the family of affinity matrices with zeros on the diagonal and off-diagonal entries
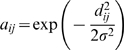
where 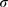 is additional parameter over which the optimization takes place. Specifically, the criterion to be maximized is
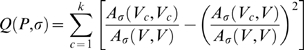
where 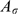 is defined as before for the new graph obtained after transformation. The 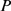 obtained in this joint maximization procedure gives the optimal modulated modularity clustering.

### MMC Implementation

Because the number of partitions 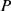 of the vertex set is large for graphs of even modest size, brute-force maximization of 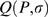 is in general not tractable. To maximize 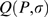 for fixed 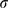, we chose to implement the divisive spectral approach of [10] because of its empirical superiority to competing approximate methods. The first step in this approach is to construct the matrix
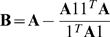
and maximize the quadratic form 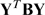 in 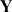. As Newman shows, the maximum modularity bipartition 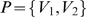 is revealed by the signs of the entries of 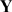 when 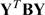 is maximized subject to the restriction that the entries of 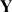 come from {+1, −1}. Upon relaxing this restriction, 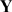 is simply the eigenvector of 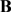 with maximal eigenvalue, and we assign the nonnegative entries of 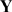 to 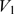. If zero is the maximal eigenvalue, the entries of 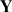 share the same sign and there exists no bipartition of the vertex set with positive modularity; otherwise, we use the Kernighan-Lin variant discussed in [10] to seek an optimal bipartition by locally refining the bipartition obtained by spectral relaxation. The end result is a bipartition 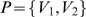, and we iterate from the first step with two new matrices 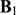 and 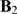 in place of 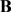. We obtain 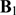 (respectively 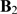) by extracting the rows and columns of 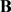 that correspond to 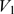 (respectively 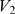) and then subtracting off the row sum from each of its diagonal entries. The approach terminates immediately with 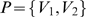 if neither 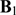 nor 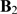 has a positive eigenvalue; otherwise, we iterate as before through the descendants of 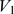 and 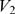 until no further bipartitions are found that increase the overall modularity.

Thus, for fixed 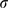 we have an approximate method for finding the 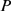 that maximizes 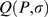. To jointly maximize 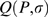 over all possible partitions 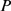 and values of 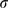, we search using the marginal maximization procedure just described over a fine grid of 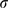 values. To expedite the joint search, we suppress the local refinement step when maximizing 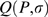 on the grid. We then choose the 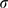 that yields the maximal value of 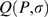 and again search over all possible partitions 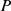, this time including the local refinement at each step. In practice, as indicated in Figure 3C, we initially bound the range of 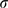 in our grid search. Our default implementation searches between 0.05 and 0.50 inclusive by steps of 0.001; this range is extended whenever the value obtained lies close to a boundary.

### Customization

In this paper, we have described and implemented a specific approach to data clustering, but the design of MMC is such that parts of it are easily modified. For example, though we have relied on a correlation-based distance to define a family of affinity matrices, our framework easily incorporates any pairwise similarity or distance metric. One possibility that has already been used for systems applications is the topological overlap metric of [8]. Given a graph described by affinity matrix 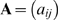 (e.g. defining 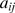 to be the absolute correlation 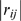), the topological overlap between nodes 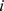 and 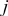 reflects their relative interconnectedness and is defined by
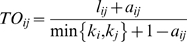
where 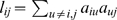 and 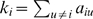 [24]. An extension to neighborhoods of arbitrary size is given in [24], and again the application is to hierarchical clustering. Hierarchical clustering is myopic by design, with each point or cluster seeing only those points closest to it. In that regard, the topological overlap metric (TOM) appears to be prescriptive by building a global relationship between points into the pairwise similarity/distance metric. By contrast, MMC is designed to be global and considers all pairwise relationships simultaneously. Thus, it is reasonable to expect that there is little to be gained by replacing our pairwise similarity metric with its TOM equivalent, and for our example datasets we found this to be the case.

Alternatively, MMC can be modified by substituting a different monotone transformation in place of our Gaussian function. This requires some care, as it is important that, when possible, the convexity of the modularity optimization surface be maintained (c.f. Figure 3C). Convexity in one dimension is inherited from modularity itself; both extremal partitions (all points share one cluster, each point has its own cluster) yield nonpositive values of 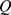, with favorable clusterings (i.e. 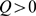) of intermediate size falling in between. In the second dimension, for our Gaussian function extreme values of 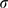 emulate the extremal partitions: a small 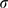 attenuates all but the strongest pairwise relationships, while a large 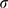 homogenizes relationship strength. Similar features result from other nonlinear functions. For example, it would be natural to parameterize a family of affinity matrices by 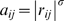, which has the advantage of subsuming the untransformed graph for 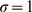. This power transformation is not so different from what we have used (see Supplementary Figure S1), and its application to our datasets yields remarkably similar results.

### Analysis of Simulated Data

We used the Matlab function *mvnrnd* to simulate 10,000 datasets consisting of nine observations drawn from a 12-dimensional multivariate Normal distribution. The distribution was specified to have mean vector zero, and each variable was specified to have marginal unit variance so that the variance-covariance matrix was equivalent to the correlation matrix shown in Figure 2. We clustered the data from each simulation using the implementation of MMC described above. The same data was subjected to average linkage hierarchical clustering as implemented by the Matlab function *linkage*; the pairwise distance metric used was the same as that used by MMC. The dendrogram produced by hierarchical clustering was always severed at the correct height to yield a prespecified number of clusters. The three spectral clustering methods to which MMC was compared were implemented in Matlab as well. The Laplacian matrices were computed as described in the Results section. Eigenvectors were obtained with the function *eig*; clusters were found using *kmeans* with squared Euclidean distance.

### Analysis of *Drosophila melanogaster* Data

As detailed in [2], whole genome variation in transcript abundance was assessed for both young males and females of each of 40 highly inbred lines using Affymetrix *Drosophila* 2.0 arrays. RNA was extracted in two independent pools of 15 flies/sex/line (40 lines×2 sexes×2 replicates = 160 samples). The raw array data was normalized using a median standardization. After normalization, an analysis of variance was used to partition variation in expression between sexes, among lines, and the sex×line interaction for each expressed transcript. At a false discovery rate of 0.001, the line term was significant for 10,096 of the expressed transcripts. A regression model identified 414 transcripts among these 10,096 that were significantly associated with the competitive fitness phenotype. The residuals from the regression model were used to compute the genetic correlations for MMC in Figure 5A. Tissue-specific expression data for each of the genes in Module 9 was obtained from [31]. The values shown in Figure 5C report the tissue-specific expression of each gene as a fraction its expression across all eleven tissues. The *doublesex* motif whose logo is shown in Figure 5D represents one of 62 *Drosophila melanogaster* transcription factors whose position-weight matrices were downloaded from http://www.bioinf.manchester.ac.uk/bergman/data/motifs/. The 5′ UTR of each gene in Module 9 was scored for the presence of all 62 motifs; our protocols for calling a motif present and for assessing enrichment are as described in [2].

### Analysis of Data from the San Antonio Family Heart Study

We used the normalized data provided by ArrayExpress under accession number E-TABM-305. Linear regressions were performed in SAS 9.1 using PROC GLM and, following [34], our model included the effects of age and sex. Gene ontology enrichment analysis was performed for each module using DAVID [36] with the list of all genes significant for the regression (673 genes) as background. Both uncorrected and corrected P-values are reported; DAVID applies the Benjamini-Hochberg procedure to correct for multiple testing.

## Supporting Information

Figure S1Comparison of monotone transformations. The absolute correlation coefficient 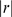 is compared to its value after transformation by each of two nonlinear monotone functions. On the left is the Gaussian function used by MMC which transforms 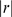 into 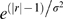. On the right is the power function 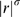. Note that the 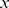-axis of the power function is coarser than that of the Gaussian function by a factor of ten.(0.14 MB PDF)
