# Supplementary material for: Modulated Modularity Clustering as an Exploratory Tool for Functional Genomic Inference
Source: PLoS Genet. 2009 May 8;5(5):e1000479. doi: 10.1371/journal.pgen.1000479 (PMC2673040; doi:10.1371/journal.pgen.1000479)
Supplement: Figure S1 — Comparison of monotone transformations. The absolute correlation coefficient is compared to its value after transformation by each of two nonlinear monotone functions. On the left is the Gaussian function used by MMC which transforms into . On the right is the power function . Note that the -axis of the power function is coarser than that of the Gaussian function by a factor of ten. (0.14 MB PDF) [file pgen.1000479.s001.pdf]

FIGURE S1

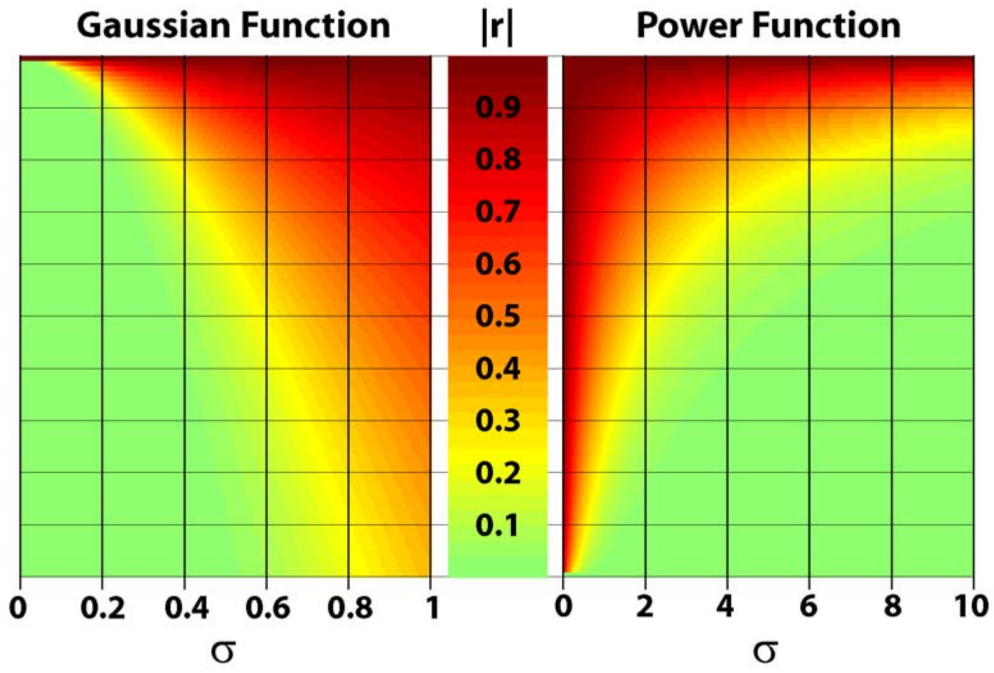

Figure S1 | Comparison of monotone transformations

The absolute correlation coefficient $|r|$ is compared to its value after transformation by each of two nonlinear monotone functions. On the left is the Gaussian function used by MMC which transforms $|r|$ into $e^{(|r|-1)/\sigma^2}$. On the right is the power function $|r|^{\sigma}$. Note that the x-axis of the power function is coarser than that of the Gaussian function by a factor of ten.
